# Power-Optimal Control of a Stirling Engine’s Frictional Piston Motion

**DOI:** 10.3390/e24030362

**Published:** 2022-03-03

**Authors:** Raphael Paul, Abdellah Khodja, Andreas Fischer, Robin Masser, Karl Heinz Hoffmann

**Affiliations:** Institut für Physik, Technische Universität Chemnitz, 09107 Chemnitz, Germany; akhodja@uni-osnabrueck.de (A.K.); andreas.fischer@physik.tu-chemnitz.de (A.F.); robin.masser@gmx.de (R.M.); hoffmann@physik.tu-chemnitz.de (K.H.H.)

**Keywords:** piston motion optimization, endoreversible thermodynamics, Stirling engine, irreversibility, friction, power, efficiency, optimization, optimal control

## Abstract

The power output of Stirling engines can be optimized by several means. In this study, the focus is on potential performance improvements that can be achieved by optimizing the piston motion of an alpha-Stirling engine in the presence of dissipative processes, in particular mechanical friction. We use a low-effort endoreversible Stirling engine model, which allows for the incorporation of finite heat and mass transfer as well as the friction caused by the piston motion. Instead of performing a parameterization of the piston motion and optimizing these parameters, we here use an indirect iterative gradient method that is based on Pontryagin’s maximum principle. For the varying friction coefficient, the optimization results are compared to both, a harmonic piston motion and optimization results found in a previous study, where a parameterized piston motion had been used. Thus we show how much performance can be improved by using the more sophisticated and numerically more expensive iterative gradient method.

## 1. Introduction

Stirling engines [1] are devices capable of transforming heat into mechanical work by utilizing almost any external heat source. Thus, they constitute an interesting alternative for power production in various scenarios, e.g., for waste heat or burnable waste gas utilization, or as part of electrothermal energy storage systems for renewable energies.

The most essential parts of a Stirling engine are the hot working space, the cold working space, and the regenerator. The two working spaces are thermally connected to external heat baths through heat exchangers. The volumes of the working spaces are cyclically varied over time so as to compress the gas at low temperature, heat it up with help of the regenerator, expand it at high temperature and cool it down again using the regenerator. One technical configuration to realize this is referred to as alpha-Stirling engine, where the two working spaces are in separate cylinders and their volumes are changed by independently movable working pistons.

To determine proper design parameters of a Stirling engine suitable for a specific application, parameter optimizations can be performed, see for example [2,3,4,5,6]. Then, the piston motions are often determined as harmonic functions or parametric functions representing specific piston drive mechanisms. In this theoretical study we focus on how the piston motion influences the engine performance by applying methods similar to previous optimizations of Stirling engines’ piston motions [7,8,9,10,11,12,13]. Experimental studies [14,15] have shown that power output improvements of Stirling engines are feasible through altering the piston motion.

In the current study we revisit [12], where we performed the optimization of an alpha-Stirling engine for a parameterized class of smooth piston motions. In contrast to this previous publication, we will not restrict ourselves to such a parameterized class of piston motions here, but we will use optimal control theory to obtain a more general solution of the optimal control problem. To this end we apply an indirect iterative gradient method [11] that exploits limit cycles in the state and costate problems to solve them for periodic boundary conditions.

In order to make such optimizations feasible, models with few degrees of freedom and low numerical effort are required. Stirling engine models are often categorized as first, second, and third order [16,17]. Especially in the case of the more detailed third order models, relatively large numerical effort is typically connected to the description of the regenerator. Therefore, several attempts [10,11,18,19] have been made to develop reduced-order regenerator models that constitute proper tradeoffs between accuracy and numerical effort for optimal control problems.

In the current study we use an ideal regenerator model [12] that is based on Endoreversible Thermodynamics. This model does not require additional differential equations to describe the regenerator dynamics. Instead, the regenerator is described as an endoreversible engine that instantaneously balances fluxes of particles, energy, and entropy. Hence, this model allows for a very-low-effort Stirling engine description including finite heat transfer between the working gas and the external heat baths, finite mass transfer through the regenerator as well as friction of the pistons.

## 2. Stirling Engine Model

### 2.1. Endoreversible Notation

Endoreversible Thermodynamics [20,21] is a sub-field of finite-time thermodynamics [22,23,24,25,26]. Generally, the common goal is to develop models that incorporate the system’s most dominant loss phenomena for describing performance features more accurately than reversible models. The focus typically is on providing models that can be solved either analytically or with low numerical effort so as to facilitate optimizations, obtain general results, and understand the overall irreversible system behavior. The application of finite-time thermodynamics is by no means restricted to heat engines and refrigerators [27,28,29,30,31,32,33,34,35], but various kinds of systems [36,37,38,39,40,41,42,43,44] can be considered.

In the endoreversible approach, physical systems are described as networks of reversible subsystems, which exchange extensities (entropy, volume, particles, …) and energy through reversible or irreversible interactions. Hence, in endoreversible modeling all irreversibilities are typically captured by the interactions whereas the subsystems can be described with the convenient tools of equilibrium thermodynamics. The most basic kinds of subsystems are (in-)finite reservoirs that contain extensities on the one hand, and engines, which represent ideal energy conversion devices, on the other.

A finite reservoir *i* is characterized by a state function $E(X_{i}^{\alpha })$ that determines its energy content depending on the amount of the extensities $X_{i}^{\alpha }$ contained in the reservoir. Here, the superscript α specifies the extensity, e.g., entropy $S_{i}=X_{i}^{S}$, volume $V_{i}=X_{i}^{V}$, and particle number $n_{i}=X_{i}^{n}$. The corresponding intensity $Y_{i}^{\alpha }$ follows as

(1) $$Y_{i}^{\alpha }=\frac{\partial E_{i}\left(X_{i}^{\alpha }\right)}{\partial X_{i}^{\alpha }},$$

where $Y_{i}^{S}=T_{i}$ is the temperature, $Y_{i}^{V}=p_{i}$ is the pressure, and $Y_{i}^{n}=\mu_{i}$ is the chemical potential of the reservoir. When it comes to specifying the state of the finite reservoir one has some freedom in the choice of state variables. One way is to specify all extensities.

The reservoir can have one or several contact points *r*, to each of which one interaction is attached. Through these interactions, the reservoir can take up or release extensities. According to the Gibbs relation every extensity flux $J_{i,r}^{\alpha }$ that enters ($J_{i,r}^{\alpha }>0$) or leaves ($J_{i,r}^{\alpha }<0$) the reservoir at *r* carries an energy flux $I_{i,r}^{\alpha }=Y_{i}^{\alpha }J_{i,r}^{\alpha }$. If the interaction involves several extensity fluxes (see multi-extensity fluxes [39]) then the overall energy flux at *r* is $I_{i,r}=\sum_{\alpha }I_{i,r}^{\alpha }$.

The dynamics of the finite reservoir can then for example be defined by a number of ordinary differential equations (ODEs), each describing the balance equation for one of the respective extensities

(2) $${\dot{X}}_{i}^{\alpha }=\sum_{r}J_{i,r}^{\alpha }.$$

In contrast, if the reservoir is considered as infinite, it is characterized by prescribing the full set of intensities $Y_{i}^{\alpha }$, which do not change—regardless of the size of the extensity fluxes $J_{i,r}^{\alpha }$.

As stated above, engines represent energy conversion devices. They can either operate cyclically or continuously, where we solely consider the latter here. They do not contain extensities and energy but only pass them on. Correspondingly, engines are characterized by a set of balance equations for all extensities and energy:

(3) $$0=\sum_{r}J_{i,r}^{\alpha },$$

(4) $$0=\sum_{r}\sum_{\alpha }Y_{i,r}^{\alpha }J_{i,r}^{\alpha }.$$

While the intensity values are equal at all contact points of a reservoir, with engines the intensity values generally differ from contact point to contact point so that all these balance equations are fulfilled.

Interactions are the modeling objects in Endoreversible Thermodynamics which generally capture all irreversibilities. They are characterized by balance equations for the extensities and energy, as well as by transfer laws. We will here only consider bilateral interactions that connect two subsystems. A bilateral interaction is reversible if and only if the intensity values are equal at the two connected contact points. This is achievable with infinitely fast transfer laws, making sure that small intensity differences are balanced instantaneously.

If the transfer laws are finite, the system might evolve in a way such that the intensity values at the bilateral interaction’s two contact points deviate. Then the interaction becomes irreversible: now energy and all extensities but entropy are conserved in it. Any proper definition of transfer laws must assure that entropy can only be produced and never annihilated.

### 2.2. State Dynamics

**Figure 1:**
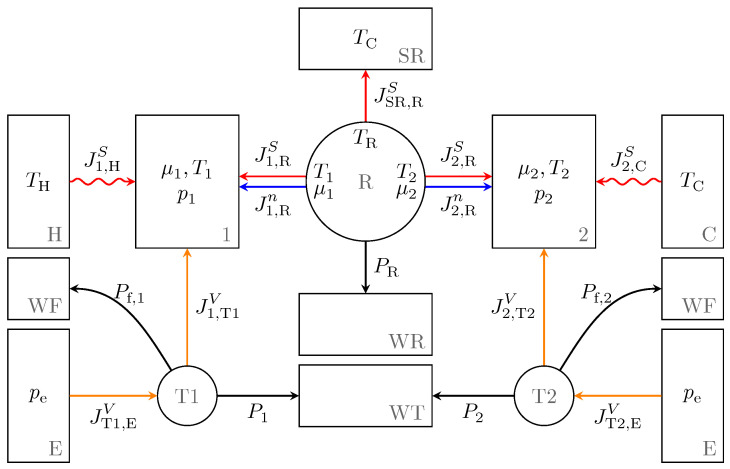
Endoreversible model of the Stirling engine with reservoirs (rectangles) and endoreversible engines (circles) as well as reversible (straight lines) and irreversible (wavy lines) interactions. On the left side the hot cylinder 1 is located with its interactions to the hot heat bath H and a transmission unit T1. On the right side the cold cylinder 2 is displayed with corresponding interactions to the cold heat bath C and a transmission unit T2. Both are connected by the regenerator R in the middle which interacts with an entropy and work reservoir, SR and WR, respectively. Further reservoirs are work reservoirs WT and WF collecting the net power and friction losses, respectively, from the energy converting engines T1 and T2 as well as volume reservoirs E representing the environment [12].

The state dynamics of the endoreversible Stirling engine model is characterized by six coupled ODEs describing the dynamics of the working space volumes, particle numbers, and entropies:

(5) $$\begin{array}{cccc} {\dot{V}}_{1} & =\frac{\nu_{V}}{\tau }\left(u_{1}\left(t\right)-V_{1}\right)\;, & {\dot{V}}_{2} & =\frac{\nu_{V}}{\tau }\left(u_{2}\left(t\right)-V_{2}\right)\;, \end{array}$$

(6) $$\begin{array}{cccc} {\dot{n}}_{1} & =\alpha \left(p_{2}-p_{1}\right)+\frac{n_{\mathrm{tot}}-n_{1}-n_{2}}{\nu_{n}\;\tau }\;, & {\dot{n}}_{2} & =\alpha \left(p_{1}-p_{2}\right)+\frac{n_{\mathrm{tot}}-n_{1}-n_{2}}{\nu_{n}\;\tau }\;, \end{array}$$

(7) $$\begin{array}{cccc} {\dot{S}}_{1} & =\kappa \left(T_{H}-T_{1}\right)/T_{1}+\frac{S_{1}}{n_{1}}{\dot{n}}_{1}\;, & {\dot{S}}_{2} & =\kappa \left(T_{C}-T_{2}\right)/T_{2}+\frac{S_{2}}{n_{2}}{\dot{n}}_{2}\;, \end{array}$$

where 1 refers to the hot working space, 2 to the cold working space, H to the hot heat bath and C to the cold heat bath, as indicated in Figure 1. The heat bath temperatures are defined as $T_{H}=400\;K$ and $T_{C}=300\;K$.

The volume dynamics from Equation (5) is defined dependent on prescribed periodic control functions $u_{i}\left(t\right)$, $i\in \left\{1,2\right\}$. Here, $\nu_{V}$ is a large number (where we will use $\nu_{V}=500$) and τ is the cycle time. This volume dynamics is similar to an over-damped mass-spring-system with moving spring support. If $\nu_{V}$ (the “spring constant”) is chosen large, then $V_{i}\left(t\right)$ will approach $u_{i}\left(t\right)$ for t→∞. This indirect way of controlling the volume through $u_{i}\left(t\right)$ allows the dynamics to approach the limit cycle independent from the chosen initial value for the volume.

The particle dynamics from Equation (6) essentially features two terms. The first term $\alpha (p_{j}-p_{i})$ describes a pressure driven particle flux between the two working spaces *i* and *j*, where α is a particle transfer coefficient. The second term was added in the current model to make sure that the particle dynamics features a limit cycle, which is essential for the application of the indirect optimization algorithm used in this study and—as above—gives freedom in choosing the initial values for the particle numbers. Here, $n_{\mathrm{tot}}$ and $\nu_{n}$ are fixed parameters, where we will use $n_{\mathrm{tot}}=1$ and $\nu_{n}=20$. For t→∞, $n_{1}+n_{2}\to n_{\mathrm{tot}}$ and then this term becomes an additive zero.

The entropy dynamics from Equation (7) is the very same as in [12]. It also features two terms, the first one describing heat transfer from or to the corresponding external heat bath with the heat transfer coefficient κ and the second one describing reversible entropy exchange with the ideal endoreversible regenerator.

The intensities in the two working spaces $i\in \left\{1,2\right\}$ can be calculated as [12]:

(8) $$\begin{array}{cc} T_{i}= & {\left(\frac{R\;T_{0}^{1+{\hat{c}}_{V}}}{p_{0}}\frac{n_{i}}{V_{i}}e^{\left(\frac{S_{i}}{n_{i}\;R}-\frac{S_{0}}{n_{0}\;R}\right)}\right)}^{\frac{1}{{\hat{c}}_{V}}}\;, \end{array}$$

(9) $$\begin{array}{cc} p_{i}= & \frac{n_{i}\;R}{V_{i}}T_{i}\;, \end{array}$$

(10) $$\begin{array}{cc} \mu_{i}= & \left({\hat{c}}_{V}\;R+R-\frac{S_{i}}{n_{i}}\right)T_{i}\;, \end{array}$$

where we use ${\hat{c}}_{V}=5/2$ for the dimensionless specific heat capacity, *R* is the ideal gas constant and $S_{0}/n_{0}$ is the working gas’s molar entropy at reference conditions $T_{0}$ and $p_{0}$. Corresponding data can for example be found in [45]. In fact, apart from constant shifts in the entropies, the system dynamics is not influenced by the definition of this reference entropy. Hence we refrain from giving values here.

Note that even though Equations (5)–(10) describe the complete system dynamics, additional care has to be taken regarding the evaluation of output quantities like mechanical power. This is due to the use of the ideal endoreversible regenerator model and will be addressed next.

### 2.3. Performance Measures

As displayed in Figure 1, the ideal endoreversible regenerator is represented by the engine R. This engine has interactions with the working spaces 1 and 2. Moreover, in order to allow for the instantaneous balancing of energy and entropy fluxes that enter or exit R at the corresponding contact points, R is given additional interactions to a heat bath SR and a work reservoir WR. The heat bath SR is chosen to have the cold heat bath’s temperature $T_{C}$. Then from the energy and entropy balances follows that

(11) $$J_{\mathrm{SR},R}^{S}=J_{1,R}^{S}-J_{2,R}^{S}$$

and

(12) $$P_{R}=T_{1}J_{1,R}^{S}-\mu_{1}J_{1,R}^{n}-T_{2}J_{2,R}^{S}-\mu_{2}J_{2,R}^{n}-T_{C}J_{\mathrm{SR},R}^{S}\;.$$

In this rather simple, ideal endoreversible regenerator model the integral surplus or deficit of the energy $\int_{0}^{\tau }P_{R}\;dt$ is then assumed to enter the overall work output:

(13) $$W_{\mathrm{out}}=\int_{0}^{\tau }p_{1}{\dot{V}}_{1}+p_{2}{\dot{V}}_{2}-\beta \left({\dot{V}}_{1}^{2}+{\dot{V}}_{2}^{2}\right)+P_{R}\;dt.$$

Here, β is the friction coefficient of the pistons. Correspondingly, the average net power output of the Stirling engine is $P_{\mathrm{out}}=W_{\mathrm{out}}/\tau$. The heat taken from the hot heat bath during one cycle is

(14) $$Q_{\mathrm{in}}=\int_{0}^{t_{0}}\kappa \left(T_{H}-T_{1}\right)\;dt$$

and the efficiency of the Stirling engine results as $\eta =W_{\mathrm{out}}/Q_{\mathrm{in}}$.

Note that detailed analyses of Stirling engines, as necessary during design development, require more detailed regenerator models than the ideal endoreversible regenerator model used here. Nevertheless, this model is considered useful when the behavior of regenerative systems is studied in a rather general manner, as it is the case here, and if additionally very low numerical effort is a key requirement.

## 3. Optimization

In this study we revisit a previous work [12] where we used a parametric optimization method to optimize the piston motion of the Stirling engine described above. This parametric optimization leads to a power-optimal piston motion that we will refer to as OS motion. In the following we will briefly introduce this parametric optimization method. Afterwards, we will describe the optimization method based on Optimal Control Theory, which we use in the present study to obtain a more general optimization result labeled COC motion.

### 3.1. Parametric Optimization (OS Motion)

In the study mentioned above [12] the piston motion of the alpha-Stirling engine was parametrized by the following function:

(15) $$V_{i}\left(t\right)=V_{\min}+\left(V_{\max}-V_{\min}\right)f\left(t/\tau ,\sigma_{i},\delta_{i}\right),$$

(16) $$f\left(t/\tau ,\sigma ,\delta \right)=f_{1}\left(f_{2}\left(t/\tau ,\sigma \right),\delta \right),$$

(17) $$f_{1}\left(y,\sigma \right)=\left(\sin\left(2\pi y+\sigma \sin\left(4\pi y\right)\right)+1\right)/2,$$

(18) $$f_{2}\left(x,\delta \right)=x+\delta \left(1-\cos\left(2\pi x\right)\right).$$

This motion is dependent on the two parameters σ and δ which can be different for the two cylinders of the engine, hence we have $\sigma_{i},\delta_{i}$ with i=1,2. The state dynamics—and consequently the power output—is thus influenced by the parameters $\sigma_{i},\delta_{i}$. Then, using an iterative optimization algorithm $\sigma_{i},\delta_{i}$ are adapted to maximize the power output. The resulting piston motion we refer to as OS motion. For more details see [12].

Note however that even though the OS motion might capture important features of the fully optimized motion, the possible shapes, which can be realized with this parametric approach, are quite limited. For example, the swept volume $\left(V_{\max}-V_{\min}\right)$ was not part of the optimization in [12]. Thus, it must be expected that the optimal power output obtained with the OS motion can be outmatched by using a more general parametrization $V_{i}\left(t\right)$.

In this study we will use Optimal Control Theory to optimize the piston motion, which will be described below. It does not require any kind of parametrization of the piston motion and will thus lead to a more general optimization result.

### 3.2. Optimal Control Theory (COC Motion)

The working volumes of the Stirling engine are here considered to result from τ-periodic control functions $u_{i}\left(t\right)$, $i\in \left\{1,2\right\}$ in terms of differential equations:

(19) $${\dot{V}}_{i}=\frac{\nu_{V}}{\tau }\left(u_{i}\left(t\right)-V_{i}\right),$$

as described in Section 2.2. Now, for prescribed cycle time τ, our goal is to choose $u_{i}\left(t\right)$ in a way such that the work output $W_{\mathrm{out}}$ is maximized for the solution of the system of ODEs

(20) $$\dot{x}=f\left(x,u\right)$$

in the time domain $t\in \left[0,\tau \right]$ under periodic boundary conditions: x(0)=x(τ). Here, we use the following definitions of the state vector $x:={\left(V_{1},V_{2},n_{1},n_{2},S_{1},S_{2}\right)}^{T}$ and the control vector $u:={\left(u_{1}\left(t\right),u_{2}\left(t\right)\right)}^{T}$ as well as the state dynamics f defined as a vector function of the latter according to Equations (5)–(10).

This constitutes a cyclic optimal control problem. To set up the necessary conditions of optimality, we define the Hamilton function:

(21) $$H:=\zeta +\lambda^{T}f\left(x,u\right),$$

where λ is a costate vector and ζ is the path target function:

(22) $$\zeta :=p_{1}{\dot{V}}_{1}+p_{2}{\dot{V}}_{2}-\beta \left({\dot{V}}_{1}^{2}+{\dot{V}}_{2}^{2}\right)+P_{R}-\mathrm{pen}\left(V_{1},V_{2}\right).$$

This is in accordance with the definition of the overall work output from Equation (13). However, here a penalty term was added in order to account for minimum and maximum volume constraints:

(23) $$\mathrm{pen}\left(V_{1},V_{2}\right):=\nu_{p0}\left(e^{\nu_{p1}\frac{V_{\min}-V_{1}}{\Delta V}}+e^{\nu_{p1}\frac{V_{1}-V_{\max}}{\Delta V}}+e^{\nu_{p1}\frac{V_{\min}-V_{2}}{\Delta V}}+e^{\nu_{p1}\frac{V_{2}-V_{\max}}{\Delta V}}\right),$$

where the prefactors are defined as $\nu_{p0}=1\;W$ and $\nu_{p1}=500$, the maximum admissible swept volume is $\Delta V=V_{\max}-V_{\min}$, and the minimum and maximum volumes are $V_{\min}=1\;L$ and $V_{\max}=11\;L$, respectively. The actual swept volume $\Delta V_{\mathrm{COC}}$ that the optimized piston paths of the COC motion involve may also turn out to be smaller than ΔV, as will be seen later.

Then the first order necessary conditions of optimality are [46,47]:

(24) $$\begin{array}{cc} \dot{x}= & \nabla_{\lambda }H\;, \end{array}$$

(25) $$\begin{array}{cc} \dot{\lambda }= & -\nabla_{x}H\;, \end{array}$$

(26) $$\begin{array}{cc} 0= & \nabla_{u}H\;. \end{array}$$

Equations (24)–(26) need to be solved for periodic boundary conditions for both the state and the costate variables: x(0)=x(τ), λ(0)=λ(τ). This is here done with an indirect iterative gradient method described in [11], which exploits the existence of attractive and repulsive limit cycles in the state problem (Equation (24)) and the costate problem (Equation (25)) for obtaining the periodic solutions.

## 4. Results

In [12] the Stirling engine piston motion was power-optimized for a parameterized class of smooth piston motions. The optimized piston motion is referred to as OS, whereas the “standard harmonic piston motion” with $V_{\min}=1\;L$, $V_{\max}=11\;L$ and 90 ${}^{\circ }$ phase shift is referred to as STD and used as a benchmark.

The alpha-Stirling engine model introduced in Section 2 is equivalent to that used in [12]. However, in the current study additional care was taken to make sure that the system dynamics feature a limit cycle in order to allow for the application of the above-mentioned optimization method. The following set of parameter values we will refer to as “reference values” in this study: $\beta_{0}=10^{5}\;Js/m^{6}$, $\alpha_{0}=100\;\mathrm{mol}/(s\mathrm{bar})$, $\kappa_{0}=\;10^{5}\;W/K$, $\tau_{0}=1\;s$. Here, the values $\alpha_{0}$ and $\kappa_{0}$ were chosen so that the associated pressures and temperatures equilibrate very fast compared to the cycle time $\tau_{0}$. Correspondingly, the associated irreversibilities are relatively small. Hence, apart from the friction irreversibility the model can be considered near-ideal. The results obtained for these values are called “reference case”. The piston motions obtained with the cyclic optimal control algorithm described in Section 3.2 will be referred to as the COC motion. In the following, we will compare it to the STD motion as well as the OS motion from [12].

**Figure 2:**
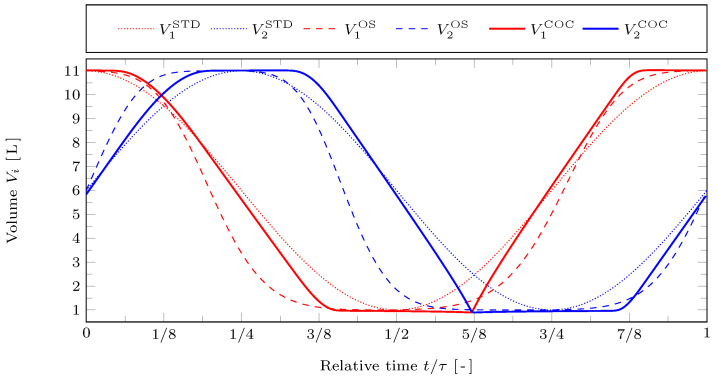
Resulting cylinder volumes $V_{1}$ and $V_{2}$ against relative time t/τ for the STD, OS, and COC motions with reference case parameters.

In Figure 2 the volumes of the working spaces are plotted against time for the reference case. The STD motion is represented by dotted lines, the OS motion by dashed lines, and the COC motion by solid lines. The red lines represent the hot working space and the blue lines the cold working space. It can be seen that there are considerable deviations between all three types of piston motions. However, the OS and the COC motion do have in common that the pistons tend to spend more time close to their bottom and top dead centers than with the STD motion. Consequently, the average piston velocities are increased compared to the STD motion. The highest piston velocity occurs for the OS motion. For the COC motion the maximum piston velocity is lower but the velocity is almost constant during four clearly distinguishable strokes. This leads to large accelerations at the transitions between those strokes, especially at t/τ≈5/8. This is connected to the fact that the path target function used here does contain friction as a function of the velocity (via ${\dot{V}}_{i}$), whereas it does not contain the acceleration (via ${\ddot{V}}_{i}$) and thus there is no penalty on fast accelerations. We will come back to this sharp transition later.

**Figure 3:**
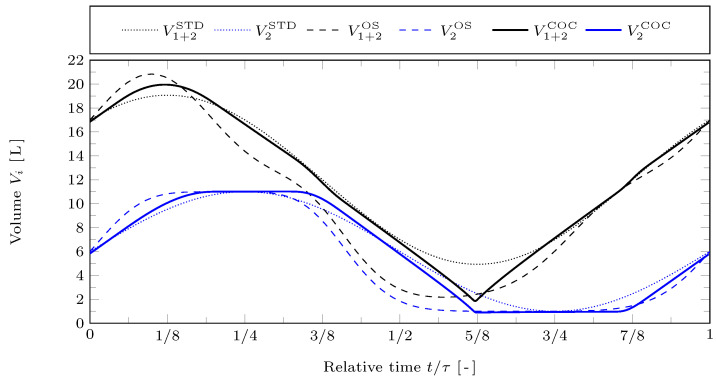
Overall gas volume $V_{1+2}$ and cold cylinder volume $V_{2}$ against relative time t/τ for the STD, OS, and COC motions with reference case parameters.

The overall gas volume $V_{1+2}:=V_{1}+V_{2}$ is depicted in Figure 3 for all three types of piston motions by black curves. The cold working volume is again shown by blue lines. As indicated above, apart from the moderate irreversibility due to friction, the reference values lead to a near-ideal thermodynamic model. It is interesting that for the considered friction law and volume constraints corresponding to an alpha-type Stirling configuration, the COC motion does not contain isochoric strokes–in contrast to the ideal Stirling cycle. Instead, the overall gas volume $V_{1+2}$ changes with approximately constant absolute rate during almost the whole cycle, in order to minimize frictional losses. This may be different for beta- or gamma-Stirling configurations.

**Figure 4:**
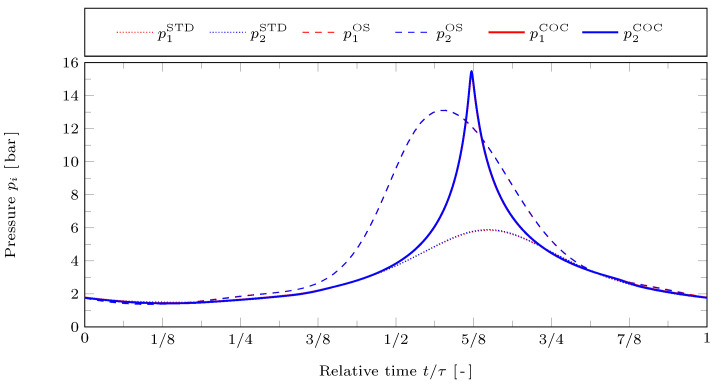
Resulting cylinder pressures $p_{1}$ and $p_{2}$ against relative time t/τ for the STD, OS, and COC motions with reference case parameters.

In Figure 4 the gas pressures in the working spaces are plotted against time for the reference case. The STD motion is represented by dotted lines, the OS motion by dashed lines, and the COC motion by solid lines. The red lines represent the hot working space and the blue lines the cold working space. Note that in this figure the blue lines lie on top of the respective red lines since there are only very small pressure differences between the two working spaces. This is due to the choice of a relatively large parameter value for the mass transfer coefficient $\alpha =\alpha_{0}$. The optimizations raise the overall difference between the minimum and maximum cycle pressures. While the minimum pressure values approximately remain the same, the OS and COC motions lead to much higher maximum pressures than the STD motion. For the COC motion the pressure curves are much more peak-shaped than for the OS motion and their maximum values are about 18% higher than that of the OS motion. With the COC motion the pressure peak occurs at the minimum of the overall gas volume $V_{1+2}$ at t/τ≈5/8, which is much lower for COC than for OS, as can be seen in Figure 3. Obviously, the shape and maximum value of that peak strongly depend on the volume constraints.

In the following we will discuss the influence of friction on the optimal piston motion and the resulting performance measures. As can be seen in Equations (13) and (22), friction is in this work modeled depending on the piston velocity in terms of $\beta \;{\dot{V}}_{i}^{2}$ with the friction coefficient β. We repeated the optimization for varying β. Here, we chose a range with $\beta \geq 0.5\times 10^{5}\;Js/m^{6}$ (for the computation of the COC motions) since for very small friction the tendency to perform more than one reciprocating piston movement in the prescribed time period grows. Correspondingly, by choosing β large enough as to prevent additional reciprocating movements, the results for the COC motion remain comparable to those for the STD and OS motions.

**Figure 5:**
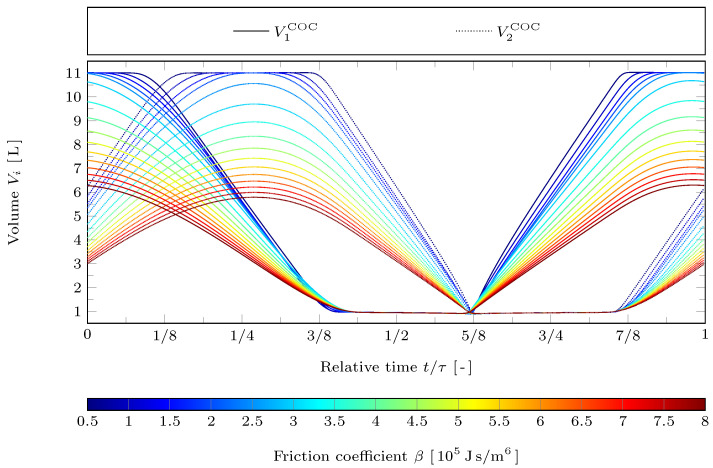
Resulting cylinder volumes $V_{1}$ and $V_{2}$ against relative time t/τ for the COC motion with reference case parameters but varying friction coefficient β.

In Figure 5 the volumes of the working spaces of the COC motion are plotted against time for varying friction coefficient β. The hot working space is represented by solid lines, the cold working space by dotted lines. Obviously, for the increasing friction coefficient the piston’s dwell times at the bottom dead center (maximum volume) reduce and above $\beta \approx 2\times 10^{5}\;Js/m^{6}$, the curves eventually detach from the maximum volume bounds. At the highest considered value of $\beta =8\times 10^{5}\;Js/m^{6}$, the swept volumes have reduced to about one half of the available volume. In contrast, the piston’s dwell times at the top dead center (minimum volume) are only slightly reduced for increasing β. Remarkably, the sharp edge at t/τ≈5/8 is not affected by increasing friction. Only the absolute values of the curvature around the volume maximums (bottom dead centers) become smaller and smaller. This behavior can be related to the path target function from Equation (22): The volumes are kept minimal as to decrease the effective dead volume and increase the pressure in the engine and thus the indicated work, which leads to the sharp edge at t/τ≈5/8. On the other hand as friction becomes more dominant the quadratic average of the piston speeds (translating to friction) are reduced while trying to achieve swept volumes (indicated work) as high as possible. This results in the rounded volume maxima.

**Figure 6:**
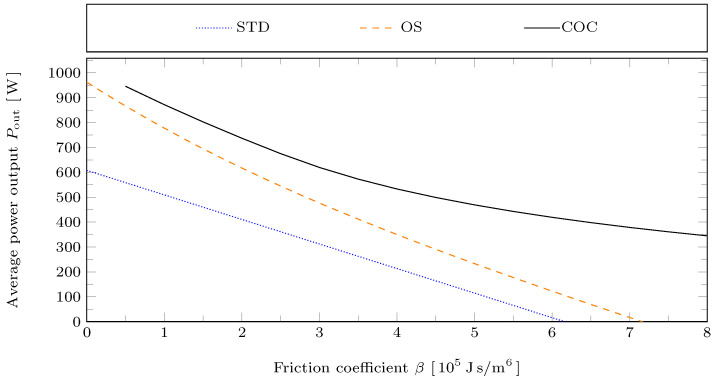
Average power output $P_{\mathrm{out}}$ for the STD, OS, and COC motions with reference case parameters but varying friction coefficient β.

From the temporal evolution of the state variables, the work output per cycle $W_{\mathrm{out}}$ is determined by integration, as defined in Equation (13). The cycle-averaged power output follows as $P_{\mathrm{out}}=W_{\mathrm{out}}/\tau$. In Figure 6 the average power output $P_{\mathrm{out}}$ of the Stirling engine is plotted against the friction coefficient β for the STD, OS, and COC piston motions. For low friction coefficient the COC motion leads to about 10% power gain relative to the OS motion. For higher values of β the power gain due to the COC motion becomes much larger than that of the OS motion. This is connected to different effects:

- STD motion: As β is increased for fixed piston motion, frictional losses increase linearly with β. Therefore, the average power output $P_{\mathrm{out}}$ decreases linearly with β.
- OS motion: As β changes, the piston motion adapts. Therefore, the average power output $P_{\mathrm{out}}$ decreases non-linearly with β. However, since the actual swept volume is fixed to the maximum admissible swept volume ΔV, the net power output $P_{\mathrm{out}}$ decays at least with a rate of $-2{\left(2\;\Delta V/\tau \right)}^{2}$. This follows from Equation (13) for the pistons moving according to a triangle wave with ${\dot{V}}_{i}=\pm \left(2\;\Delta V/\tau \right)$.
- COC motion: As β changes, the piston motion adapts not only in its shape, but also in its actual swept volume $\Delta V_{\mathrm{COC}}$. This can be seen in Figure 5. Starting from β≈2 the actual swept volume $\Delta V_{\mathrm{COC}}$ continuously decreases as β is increased. Therefore, now the lower bound for the rate of decay of $P_{\mathrm{out}}$ is only $-2{\left(2\;\Delta V_{\mathrm{COC}}/\tau \right)}^{2}$, which quadratically reduces with $\Delta V_{\mathrm{COC}}$. Correspondingly, it can be seen in Figure 6 that the decay of $P_{\mathrm{out}}$ with increasing β is much slower for the COC motion.

**Figure 7:**
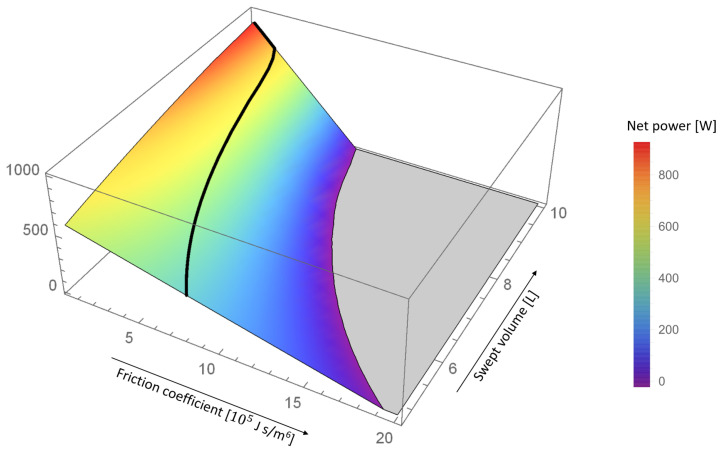
The thick black line represents the average power output $P_{\mathrm{out}}$ of the COC motion plotted against the friction coefficient β and the actual swept volume of the cold piston (see Figure 5). The color surface was obtained by varying β, while leaving the swept volume and the shape of the piston motion fixed. Correspondingly, the swept volume here is a proxy for the shape of the piston motion from Figure 5. The plot range is restricted to values above zero watts.

To make the latter point clearer we plot the optimization result of the COC motion already shown in Figure 6 (black line) against both the friction coefficient β and the actual swept volume (of the cold piston) in Figure 7. The COC motion is here represented by the thick black line. The color surface apart from that line was obtained by varying β, while leaving the swept volume and the shape of the piston motion fixed. That is, in Figure 7 the swept volume is a proxy for the shape of the piston motion according to Figure 5.

It can be seen that, starting in the upper corner, the net power output is reduced by both increasing the friction coefficient and decreasing the swept volume. If the swept volume is held constant as β is increased, the decay of net power is very strong, not unlike it happens for the STD and OS motions. The COC motion (thick black line), however, avoids this strong decrease by reducing the swept volume for larger β.

**Figure 8:**
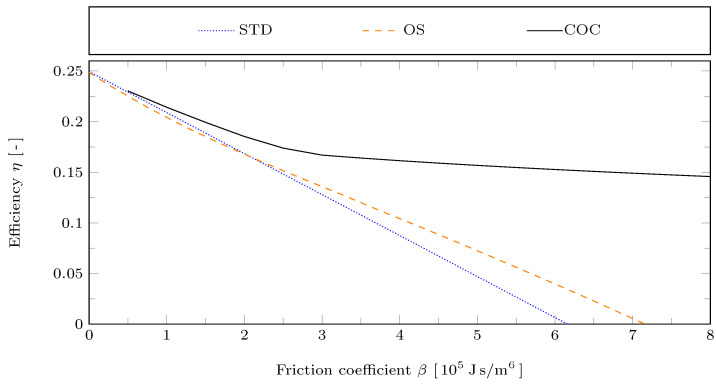
Efficiency η for the STD, OS, and COC motions with reference case parameters but varying friction coefficient β.

The influence of the friction coefficient β on the Stirling engine’s efficiency is shown in Figure 8. For small β the efficiency approaches a value of 0.25 for all piston motions, which corresponds to Carnot efficiency. This is because the reference values $\kappa_{0}$ and $\alpha_{0}$ of the heat and mass transfer coefficients were chosen relatively large, so that the corresponding irreversibilities are negligible.

The optimizations of the piston motion were performed for the target function being the power output in both cases, with the OS and the COC motion. Therefore, the efficiency resulting from the optimized motions will not necessarily be lager than that of the STD motion. In fact, for the OS motion it can be observed that its efficiency goes below that of the STD motion for β below about $2.1\times 10^{5}$ Js/m^6^. For the COC motion this does not occur in the considered range with $\beta \geq 0.5\times 10^{5}\;Js/m^{6}$. For large β both, the OS and the COC motion lead to increased efficiency. However, the efficiency increase due to the COC motion is much more significant, which is again partially related to the reduction in the swept volumes.

## 5. Conclusions

In this study we applied cyclic optimal control theory to power-optimize the piston motion of an alpha-Stirling engine with dominating mechanical friction irreversibility. The underlying endoreversible Stirling engine model additionally takes finite heat and mass transfer into account. However, we here used large transfer coefficients so that, apart from friction, a near-ideal thermodynamic model was obtained. The optimizations were repeated for the varying friction coefficient. The results for the optimized piston motions (COC) were compared to results of a previous study [12], where parameterized piston motions had been optimized (OS). Moreover, harmonic piston motions (STD) were resorted to as a benchmark.

The optimized piston motions OS and COC lead to increased pressure variations during the cycle, which bring about significant gains in power. The COC motion obtained with cyclic optimal control theory in the current study, outmatches the OS motion from [12] regarding both power and efficiency. This especially holds true for high values of the friction coefficient. However, in the COC motion considerably higher accelerations of the pistons occur. This is connected to the used definition of frictional losses involving only the piston velocities, not accelerations.

An interesting result is that for given engine parameters, there is a certain swept volume for which net power becomes optimal. Increasing swept volume beyond this value would result in reduction of net power.

Moreover, it was shown that for the considered friction law and volume constraints corresponding to an alpha-Stirling configuration, the COC motion does not contain isochoric strokes, which is in contrast to the ideal Stirling cycle. For other Stirling configurations this might however be different.

For more detailed analyses, as required in engineering, additional subsystems as well as transfer and friction laws describing a specific Stirling engine design can be included in the model. Moreover, in this case a more detailed irreversible regenerator model should be used. A low-order endoreversible regenerator model developed for this purpose is, for example, described, numerically validated and applied in [11].
